# Upward and Poleward (but Not Phenological) Shifts in a Forest Tenebrionid Beetle in Response to Global Change in a Mediterranean Area

**DOI:** 10.3390/insects15040242

**Published:** 2024-03-30

**Authors:** Simone Fattorini

**Affiliations:** Department of Life, Health and Environmental Sciences, University of L’Aquila, Via Vetoio, 67100 L’Aquila, Italy; simone.fattorini@univaq.it

**Keywords:** *Accanthopus velikensis*, Apennines, Coleoptera Tenebrionidae, global warming, insect conservation, Italy, mountains

## Abstract

**Simple Summary:**

There is increasing interest in the study of how the ongoing climate change is affecting insect populations. However, most research has been focused on a limited number of groups that are particularly charismatic or expected to respond more clearly to climate change. Moreover, most research has been developed in a few areas (especially northern and central European countries), while others (such as the Mediterranean basin) have been largely overlooked. Therefore, more empirical research is needed, particularly on less charismatic species, groups that are expected to react less dramatically to climatic change, and key areas that are still poorly investigated. This study investigates changes in distributional and activity patterns in a common, ecologically tolerant, forest tenebrionid beetle in a region (central Italy) within the Mediterranean biodiversity hotspot, an area strongly subjected to the ongoing climate change, but for which research is still limited. By analyzing changes in elevation, latitude, longitude, and seasonal activity between 1900–1980 and 1981–2022, the results provided insights into the potential effects of climate change on this species. The beetle’s average values of elevation and latitude were found to have been increased in the second period. In response to rising temperatures, the species became more frequent at higher elevation and in northern places. No strong evidence was found for an earlier species seasonal activity, but this could be due to the inclusion in the analysis of likely overwintering individuals. The findings suggest that even thermally tolerant species can undergo distributional shifts in elevation and latitude, even at a relatively small scale.

**Abstract:**

There is an increasing volume of literature on the impact of climate change on insects. However, there is an urgent need for more empirical research on underrepresented groups in key areas, including species for which the effects of climatic change may seem less evident. The present paper illustrates the results of a study on a common forest tenebrionid beetle, *Accanthopus velikensis* (Piller and Mitterpacher, 1783), at a regional scale within the Mediterranean basin. Using a large set of records from Latium (central Italy), changes in the median values of elevation, latitude, longitude, and phenology between two periods (1900–1980 vs. 1981–2022) were tested. Records of *A. velikensis* in the period 1981–2022 showed median values of elevation and latitude higher than those recorded in the first period. Thus, in response to rising temperatures, the species became more frequent at higher elevation and in northern places. By contrast, *A. velikensis* does not seem to have changed its activity pattern in response to increased temperatures, but this might be an artifact due to the inclusion of likely overwintering individuals. The results obtained for *A. velikensis* indicate that even thermally euryoecious species can show changes in their elevational and latitudinal distribution, and that poleward shifts can be apparent even within a small latitudinal gradient.

## 1. Introduction

Due to anthropogenic causes, the ongoing climate change is occurring at a rate unprecedented in the history of Earth, representing one of the most important drivers of ecological change worldwide [1,2,3,4]. Although the impacts of climate change on species distributions may show considerable variation (including no or counterintuitive effects) [5,6,7,8,9,10,11], the most commonly documented patterns in response to increasing temperatures are range expansions/shifts upslope (i.e., to higher elevations) and poleward (i.e., to higher latitudes) [9,10,12,13,14,15,16,17,18,19,20,21,22,23,24,25,26,27,28,29,30]. Species that cannot track their thermal niches through upslope and poleward movements because of their low dispersal capabilities or geographical constraints (e.g., species that are already on mountain tops cannot move further uphill), and that cannot adapt to the new climatic conditions, are subject to population decline and even local or global extinction if their environment becomes climatically unsuitable [30,31,32,33,34,35,36]. As seasonal activities such as breeding and flowering are profoundly influenced by climatic conditions, climate change also affects species phenology (i.e., the timing of seasonal events in their life cycles) [37,38]. For example, several studies have documented earlier flowering in many plant species in response to rising temperatures [10,39,40,41] and many birds have advanced their breeding phenology [10,42,43,44] and spring migration [45]. The advancement of phenology has also been observed in other vertebrates, such as amphibians [10,46], reptiles [10,24], and mammals [47], and in invertebrates [10,15,43,48,49].

There is an increasing amount of literature on the impact of climate change on insect distribution and phenology. Empirical evidence indicates that, with increasing temperatures, insects tend to track their climatic niche by moving upslope [10,17,28,30,35,50,51,52,53,54,55,56,57,58,59,60] and poleward [17,50,61,62,63,64,65,66,67,68,69,70,71]. Changes in phenology are also documented in many insects, with a shift toward earlier seasonal activity being the most commonly recorded pattern [38,49]. However, current studies on insect responses to climate change suffer from many limits: (1) empirical studies have been progressively outweighed by predictive work [28,30,36,72,73,74,75,76,77,78,79,80,81,82,83], which has led to a substantial scarcity of empirical data; (2) most work has been focused on a few, usually charismatic taxa such as butterflies and dragonflies [19,30,35,51,52,55,59,60,61,65,66,68,71,73,84,85], while responses in most insect taxa remain unknown; (3) most research has been conducted in relatively few areas, with a strong preponderance of studies conducted in Europe, especially in central and northern countries [9,10,17,23,26,50,53,59,60,61,68,70,71,86,87], while the impacts in other contexts remain poorly investigated; (3) attention has mostly been paid to species expected to respond more dramatically to climate change (e.g., thermal specialists living at high elevations and latitudes), while responses in species assumed to be less vulnerable/responsive are overlooked [28,30,32]; (4) research on latitudinal shifts has been focused on relatively large scales, usually at country level or even at broader scales [17,23,50,61,68,70,71,87], while responses at smaller scales are substantially unexplored. Given the enormous diversity of insects, this calls for more empirical research expanding the taxonomic, geographical, and ecological scope of current work on the impact of climate change on these animals. In particular, there is an urgent need for more empirical research on underrepresented groups in key areas, including species for which the effects of climatic change may seem less evident.

To contribute toward reducing these gaps, the present paper illustrates the results of an empirical study on the effects of climate change on a generalist, forest tenebrionid beetle, *Accanthopus velikensis* (Piller and Mitterpacher, 1783), at a regional scale within the Mediterranean basin.

The Mediterranean basin is one of the global hotspots of biodiversity [88,89,90,91] whose conservation is strongly threatened by the global change. The Mediterranean region is warming 20% faster than the global average, and precipitations are expected to drop dramatically (with 2 °C global warming, precipitation will be reduced by ~10 to 30% and maximum daytime temperatures will likely increase by 3 °C; 4 °C of global warming will make nearly all nights tropical, with almost no cold days) [92].

Tenebrionids are one of the largest families of Coleoptera, comprising about 30,000 known species [93]. Most tenebrionids (both adults and larvae) are saprophagous insects, feeding on a variety of decaying matter [94,95,96]. Several species, however, feed on fungi, algae, lichens, and mosses; a few are predators or semipredators feeding on other insects; and many ground-dwelling larvae feed on living plant roots, stems, or seedlings [94,95,96]. Tenebrionids occur in the most diversified ecosystems: although these beetles are mostly known to be particularly speciose in arid and semiarid environments, being a conspicuous component of coastal and desert faunas, many species are associated with forests [94,95,96,97,98].

*Accanthopus velikensis* (also known as *Enoplopus dentipes* (Rossi, 1790), especially in the past, and sometimes as *Enoplopus caraboides* (Petagna, 1786) in the older literature) is a European tenebrionid distributed in mainland Italy (Piedmont [99], Lombardy [99,100,101,102,103], Trentino-Alto Adige [99,104], Veneto [99,104], Friuli-Venezia Giulia [99,105], Liguria [106], Emilia-Romagna [99,107,108], Tuscany [109,110,111,112], Umbria [113], Marche [114,115,116], Latium [117,118,119,120,121,122,123,124,125,126,127,128,129,130,131,132], Campania [115,133], Abruzzi [134,135], Molise [136], Basilicata [115,137,138,139,140,141,142], Calabria [113,115,133,142,143,144,145,146], Apulia [115,133,142,147,148]), Sicily [115,149,150,151], Sardinia [152], Elba Island [153], Salina Island [154], southwestern France (Alpes-de-Haute-Provence, Alpes-Maritimes, Var) [155,156,157,158], Corsica [158,159], Switzerland [159,160,161], Albania [162], Slovenia [105], Croatia [113,160,162], Dalmatian Islands [105,160], Bosnia-Herzegovina [113,162], Montenegro [113,160,162], Serbia [163,164], Greece [165,166,167,168], Bulgaria [169,170,171,172,173], Romania [162,174,175,176], Poland [177,178,179], Hungary [180,181], and European Turkey [182,183]. Köhler and Klausnitzer [184] indicate its presence in Germany as erroneous, but citations from some places in Bavaria (Munich environs, Landsberg am Lech, Wildbad Kreuth, Wasserburg am Inn) are authoritative [137]. A citation from Austria [185] might refer to areas that were once within the Austrian territory, but that belong now to Croatia or Italy; thus, the presence of this species in Austria is possible [186], but needs confirmation [182]. The species is also generically quoted from North Macedonia [187].

The species has wide ecological preferences and is found in a variety of forest types, especially broadleaves, from the sea level to about 1200 m elevation, being associated with beeches (*Fagus sylvatica*) [104,133], chestnuts (*Castanea sativa*) [133,158], oaks (*Quercus* sp.) [104,119,133], cork oaks (*Quercus suber*) (Fattorini, pers. obs.), birches (*Betula*) (Fattorini, pers. obs.), elms (*Ulmus* sp.) [133], hazels (*Corylus avellana*) [104], plane trees (*Platanus* sp.) [153], silver firs (*Abies alba*) [110], Aleppo pines (*Pinus halepensis*) [158], Calabrian pine (*Pinus nigra laricio*) [133], eucalypts (*Eucalyptus* sp.) [125], and acacias (*Acacia* sp.) [125]. In Friuli-Venezia Giulia, it is reported as common in humid deep valleys with mixed broadleaved forests around the city of Trieste [105]. In Tuscany, it has also been found in wet zones [111]. In Apulia, it has been found in an ecotonal zone between maquis and broadleaved forests, in beech forests, and in pinewoods [148]. In Latium, it has been collected in hygrophilous woods [119,121], holm oak forests [119], pinewoods [122], beechwoods, chestnut groves, mixed forests, and Mediterranean maquis (Fattorini, pers. obs.). In northern Greece, Schawaller [167] found the species in a *Fagus*/*Quercus* forest, a *Quercus*/*Carpinus* forest, and in a bush forest with *Quercus*, *Castanea*, and *Fagus*. In Bulgaria, it was found in large numbers in chestnut forests [172], and it was also commonly found in the chestnut forests of Alban Hills (Latium) (Fattorini, pers. obs.). In Turkey, it was found in a *Fagus*/*Quercus* forest [182]. In France, it was reported to occur in small humid valleys [158], and, in Corsica, it was found in a riparian forest rich with chestnuts [159].

The species (which is indicated as feeding on lichens [183]) is usually found under bark [104,120,158,167], but it can be also found in a variety of other microhabitats, including under stones [108,120,159,160], in the debris of old trees [160], in the litter of forest habitats (Fattorini, pers. obs.), on hedges [160], in tree holes (*Abies alba* [110]), and even in carcasses [188].

The species is nocturnal and frequently gregarious, which can lead to many torpid and inactive individuals being aggregated in the same microhabitat during the day; at sunset, they become active, walking on the trees or at their base [119]. Adults of *A. velikensis* can be observed all year round [119,121,158]. In central Italy, it is usually present in large numbers, being one of the most abundant tenebrionids of forest communities, even in fragmented landscapes, including urban green spaces [120,121,123,125,126,127,131,189]. *A. velikensis* is, therefore, a species with a wide geographical distribution, a broad elevational range, wide ecological preferences, and usually abundant populations. These characteristics make it an excellent model organism to investigate the effects of climate change on generalist forest beetles.

I focused on the response of *A. velikensis* to climate change in central Italy based on data collected from the Latium region, as this area has been intensively sampled by entomologists since the beginning of the 20th century [124]. To test whether the ongoing climate change impacted *A. velikensis* ecology, I divided the data into two periods: a first period (1900–1980), which should reflect climatic conditions before the effects of the ongoing climate change, and a second one (1981–2002) that was characterized by increased average temperatures. Because of the opportunistic nature of the data, I focused on the spatial and temporal frequency distribution of *A. velikensis* records, as the sampling sites differed between periods and the use of numbers of individuals would have been more strongly subject to collecting biases (for example, the use of pitfall traps in certain sites in some years might have led to the collection of very large numbers of individuals, compared to those commonly collected by hand searching).

On these bases, I hypothesized that—as a consequence of increasing temperatures—in the second period, *A. velikensis* became more frequent at higher elevations (H1) and at higher latitudes (H2). Moreover, I hypothesized that the species advanced its phenology (H3) in the second period.

These hypotheses led to the following predictions:

1. Prediction 1: Based on H1, the average elevation records of *A. velikensis* should be higher in the second period.
2. Prediction 2: Based on H2, the average latitude records of *A. velikensis* should be higher in the second period.
3. Prediction 3: If changes predicted by Predictions 1 and 2 are really due to climate change and not to increased sampling at higher elevations or latitudes in the second period, the localities in the second period should have current temperatures similar those that characterized the localities in the first period (isotherm tracking hypothesis [11]). If we assume that the species did not change its thermal optimum between the two periods, in the second period, it should have been recorded more frequently in localities that, based on their position (elevation and latitude), have, on average, temperatures similar to those that the species experienced in the localities from which it was found in the first period, and which are now less suitable because of temperature increases. If, on the contrary, the records from the second period were characterized by substantially lower temperatures than those of the first period, this would indicate that the changes in the species’ average elevation and latitude were biased by increased sampling at higher elevations and latitudes. In this case, the results in accordance with Prediction 1 and 2 would not necessarily support H1 and H2.
4. Prediction 4: If the changes predicted by Prediction 1 are really due to climate change and not to increased sampling at higher elevations, no longitudinal effect should be deduced. As the mountain areas prevail eastward, a significant increase in records eastwards would indicate that a higher frequency of records from higher elevations might have been biased by increased sampling in more mountainous sectors of the study area.
5. Prediction 5: Based on H1 and H2, in the second period, *A. velikensis* should have been more frequently found in localities with phytoclimatic conditions indicating lower temperature and lower aridity. Phytoclimatic approaches classify areas integrating climatic and vegetational characteristics [190]. Using a phytoclimatic classification based on data for the first period, and assuming that conditions have now changed, in the second period, *A. velikensis* should have been recorded more frequently in phytoclimatic units that were originally classified as expressing colder and more humid conditions.
6. Prediction 6: Based on H3, the frequency of records in the second period should exhibit an advanced average value for the month or day of collection.

## 2. Materials and Methods

### 2.1. Study Area

**Figure 1:**
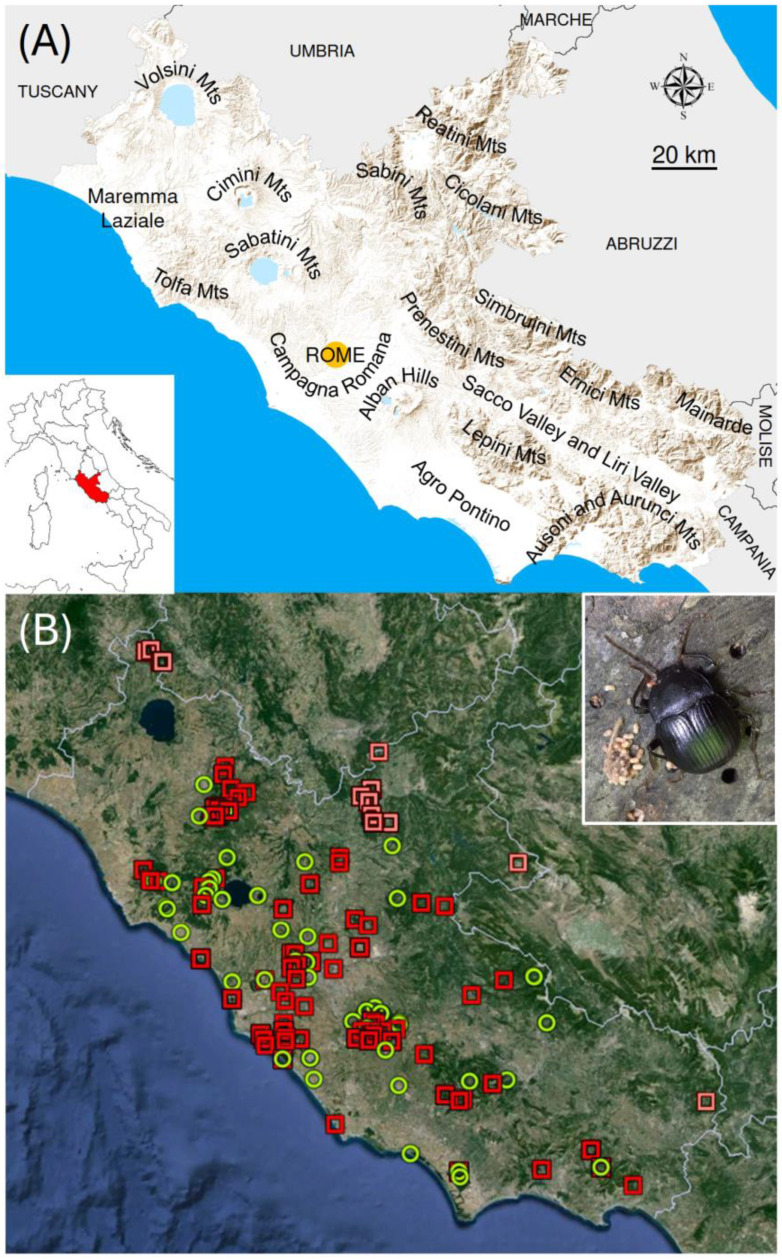
Study area (Latium, central Italy). (**A**) Main orographic systems. The inset shows the location of Latium (in red) within the Italian territory. (**B**) Distribution of records of *Accanthopus velikensis* (each dot is a locality for which one or more records are available). Circles: Data for the period 1900–1980. Squares: Data for the period 1981–2022. Pink squares indicate data omitted from the analyses conducted with the reduced dataset. Image generated using Google Earth Pro (version 7.3.6.9750). The inset shows an adult individual of *A. velikensis* in its habitat.

The study involved the distribution and phenology of *Accanthopus velikensis* in Latium (central Italy) (Figure 1). This region extends for 17,232 km^2^, of which 20% is flat, 54% hilly, and 26% mountainous [191]. Coasts are mainly represented by sandy beaches (with sparse relicts of dune vegetation), with rocky coasts being limited to small areas in the southernmost part of the region. Coastal plains are represented by the Maremma Laziale in the northern sector of the region (a formerly mostly malarial marshland), the Campagna Romana in the middle (a large alluvial plane whose characteristics reflect the millenary agricultural and pastoral activities associated with the presence of the city of Rome), and the Agro Pontino in the southern part (another formerly swampy area that was largely reclaimed to extirpate the malaria). These lowland areas are mainly occupied by cultivated fields, interspersed with remnants of natural vegetation mostly represented by Mediterranean shrublands and maquis, with rare fragments of hygrophilous woodlands. The mountainous territory of the region includes both pre-Apennine and Apennine mountains. The pre-Apennines comprise groups of mountains of volcanic origin (Volsini, Cimini, Tolfa, and Sabatini mountains), mainly characterized by mixed broadleaved forests; the Alban Hills (also of volcanic origin), which are characterized by chestnut (*Castanea sativa*) woods; and, in the southern part, the Lepini, Ausoni, and Aurunci Mountains (calcareous), where typical evergreen broadleaves (*Quercus ilex* and *Quercus suber*) prevail. The Apennines of Latium are a continuation of the Apennines of Abruzzo, and include the Reatini, Cicolani, Sabini, Prenestini–Tiburtini–Ruffi, Simbruini–Catari, and Ernici–Mainarde Mountains, which are mainly characterized by the presence of deciduous oak woods (*Quercus cerris*, *Quercus pubescens*) and beech woods (*Fagus sylvatica*). For further details on the geography and vegetation of Latium, see, for example, [192,193,194,195]. Because of its complex topography (the highest peak is Mount Gorzano at 2458 m, on the border with the Abruzzi region), the region shows a wide variety of climates, ranging from typically Mediterranean climates along the coasts (with average annual temperatures around 17 °C, less than 1000 mm of annual precipitation, and a distinct period of summer aridity) to subalpine climates on the highest peaks (with average annual temperatures < 10 °C, about 1500 mm of annual precipitation, and a lack of a period of summer aridity) [190,196].

### 2.2. Data Sources

The data used in this paper were mainly obtained from the examination of material preserved in public and private collections (1409 examined specimens) supplemented with literature information, for a total of 404 records. I considered any data that differ in terms of location, elevation, and/or date of collection as separate records. These data spanned from 1900 to 2022 and, because of the variety of sources used, they form a random sample not affected by biases due to collector preferences for certain areas or habitats. The insects were collected using a variety of methods, including hand searching (on the ground, under stones and fallen trees, on trees, under bark, etc.), pitfall traps, Malaise traps, and soil examination (with sand and litter sieving), in both open and forest vegetation from sites distributed through the whole elevational gradient of the species.

Where label data included elevation and geographical coordinates, I used this original information for the analyses. Otherwise, I deduced the elevation and geographical coordinates of records from locality names reported on the label with the maximum allowed precision. For material obtained from pitfall traps, if the period of trap activity was between two months, I assumed that individuals were collected during the month in which the trap was more active (for example, individuals collected from 24 September to 24 October 1985 were assumed to be collected in October 1985). Julian calendar dates (1 January = day 1, etc.) were calculated only when the day of collection was known. Each record was assigned to a phytoclimatic unit using the classification proposed by Blasi [190]. These phytoclimatic units integrate information on climate and vegetation using data on average monthly rainfalls, averages of minimum and maximum monthly temperatures, and plant (mostly tree) associations. Following the original scheme, the phytoclimatic units were numbered from 1 to 14 reflecting increasing temperatures and aridity (unit 15 was exclusive to the Pontine Islands, and, hence, absent in the study area; Table S1). All data are reported in Table S2.

To depict variations in the average annual temperatures from 1990 to 2022 over the study region, I used data from [197] (Table S3, Figure S1). To model variations in the annual temperatures in response to elevation, latitude, and longitude in the study area, I used a dataset of climatic data for the period 1955–1985 [190] (Table S4). This dataset included data from 108 meteorological stations, from which I excluded those of Ponza and Pratolungo as they were from an insular station and a locality out of the Latium region, respectively. As for the elevation of the meteorological stations, I used those reported by Blasi [190]. The coordinates of the meteorological stations were deduced from their location, as shown in the map provided in this reference, because they were not explicitly given.

### 2.3. Data Analysis

To assess shifts in the frequency distribution of elevation, latitude, longitude, phytoclimate, month, and day of *A. velikensis* records, I divided the data into two periods: 1900–1980 and 1981–2022. I adopted this division because the rapid climate warming in Italy in general and in the study area in particular started around the 1980s [198,199,200,201] (see Figure S1). I used a one-tailed *t*-test with correction for unequal variances to test the significance in the difference in the average annual temperatures between the two periods using the data from Table S3.

Differences in the median values of elevation (Prediction 1), latitude (Prediction 2), longitude (Prediction 4), phytoclimate (Prediction 5), and month and day (Julian date) (Prediction 6) in *A. velikensis* records between the two periods were tested using one-tailed Wilcoxon rank sum tests with continuity correction. For phenology, I used both month and Julian date, as, for many records, only the month of collection was known.

To test Prediction 3, I modeled how the average annual temperatures in the study area varied with elevation, latitude, and longitude using a multiple linear regression with the data given in Table S4. Based on the multiple regression model with these climatic data, I calculated the thermal optimum of *A. velikensis* for the first and second periods by introducing into the model the values of median elevation, latitude, and longitude of *A. velikensis* records calculated for the two periods, respectively. Then, the resulting optima were compared, assuming an increase of 0.9 °C (as this was the average increase observed in the study region—see Results) in all localities for the second period. If, taking into account this correction, the two optima were similar in the two periods, changes in the median values in the distribution of records in the second period are assumed to reflect an increasing frequency of collection in places that were close to the thermal optimum of the species, as deduced from the data from the first period.

Because of the opportunistic nature of the data, they might be affected by some biases toward higher elevations and latitudes, if sites at higher elevations and latitudes were more accessible in the second period. To reduce this risk, I repeated the analyses by excluding records for the second period that were located in areas far from any records of the first period.

All statistical analyses were performed in R [202] with the packages stats and ggplot2. In all cases, statistical significance was set at α = 0.05. Figure 1A was constructed in ArcGIS Pro 3.1.3 [203] with the function Hillshade using data from TINITALY [204].

## 3. Results

The mean of the average annual temperatures for the period 1981–2022 (14.12 °C) was significantly higher than the mean for the period 1900–1980 (13.23 °C) (*t* = 8.904, df = 62.543, *p* < 0.0001), with an average increase of 0.89 °C.

A multiple regression model indicated that the average annual temperatures decreased with elevation and latitude, whereas longitude did not have a significant effect (Table 1).

**Figure 2:**
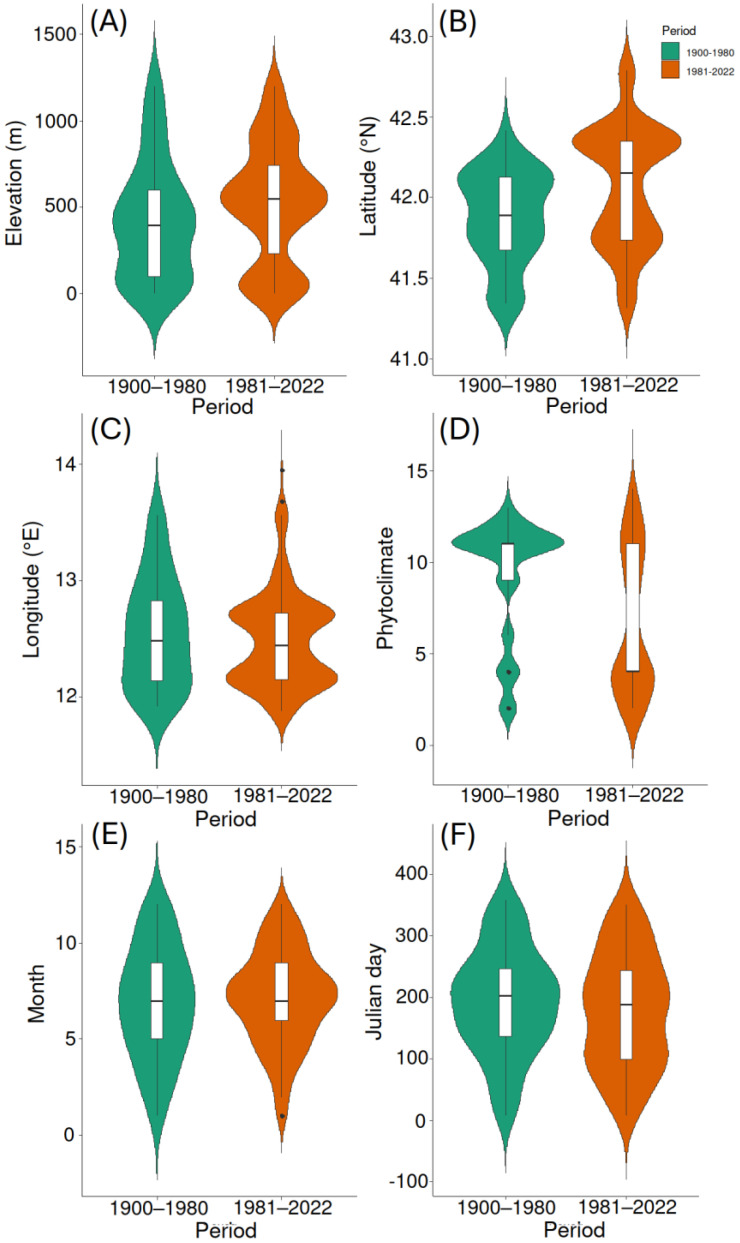
Violin plots illustrating changes in elevation (in m) (**A**), latitude (° N) (**B**), longitude (° E) (**C**), phytoclimate (increasing values indicate warmer and drier climates) (**D**), month (**E**), and day (Julian date) (**F**) of records of the tenebrionid *Accanthopus velikensis* in Latium (central Italy) in two periods (1900–1980 and 1981–2022). Kernel densities, medians, quartiles, ranges, and outliers are represented. Results obtained with the full dataset.

The records of *Accanthopus velikensis* changed their median elevation from 393 m in the period 1900–1980 to 550 m in the period 1981–2022 (*W* = 10,278, *p* = 0.002) (Figure 2A). This means an uphill shift of about 157 m between the two periods. Using the mid-points of the two recording periods (i.e., 1940 and 2002, respectively; hence, a period of 62 years), this corresponds to an average upward shift of about 2.5 m per year.

A significant change was also observed in the median latitude, from 41.892° N in 1900–1980 to 42.153° N in 1981–2022 (*W* = 8610, *p* < 0.0001) (Figure 2B). This means a northward shift of about 0.26° latitude (i.e., about 30 km) of the median (which means an average poleward shift of 484 m per year).

No significant change was detected for the median longitude between the first period (12.490° E) and the second period (12.447° E) (*W* = 13,312, *p* = 0.769) (Figure 2C).

Based on the multiple regression model with climatic data for the 1955–1985 period, the thermal optimum of *A. velikensis* in the first period (i.e., for an elevation of 393 m, a latitude of 41.892° N, and a longitude of 12.490° E) was an average annual temperature of 14.16 °C, whereas for the second period (i.e., for an elevation of 550 m, a latitude of 42.153° N, and a longitude of 12.447° E), it was 13.43 °C. This means an increase of records in the second period from localities that (in 1955–1985) were, on average, 0.7 °C colder than those of the first period. These localities are expected to be now, on average, 0.9 °C warmer than in the 1955–1985 period used as reference. Thus, the true thermal optimum of *A. velikensis* in the second period would be estimated at around 14.3 °C (i.e., 13.4 + 0.9 °C), which is extremely close to that calculated for the first period (14.2 °C). This suggests that changes in the median values in the distribution of records reflected an increasing frequency of collection in places that were close to the thermal optimum of the species.

*Accanthopus velikensis* also showed a significant increase in records from localities with colder phytoclimates in the second period (*W* = 16,554, *p*-value < 0.00001) (Figure 2D). In the first period, the median value corresponded to the phytoclimatic unit 11, whereas the median value in the second period corresponded to the phytoclimatic unit 4. This means that, in the second period, the species was more frequently collected in habitats characterized by vegetation associated with colder and more humid climates. Overall, the species was not recorded from phytoclimatic units 1, 5, and 8.

No significant change was detected for the median month between the first and the second period, which was July in both cases (*W* = 11,652, *p* = 0.760) (Figure 2E). A certain shift toward earlier dates in the frequency of records was observed in the second period, as the median was 22 July in the first period and 7 July in the second period, but the difference was not significant (*W* = 2926.5, *p* = 0.145) (Figure 2F).

**Figure 3:**
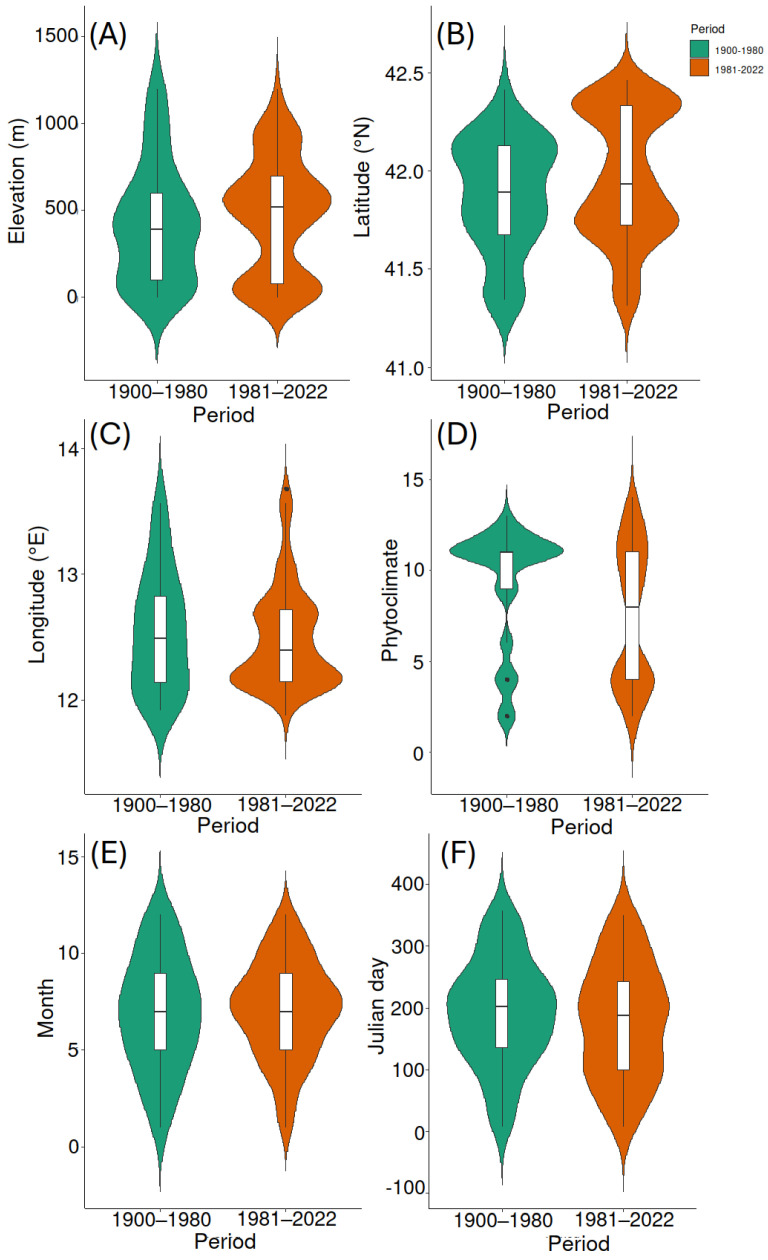
Violin plots illustrating changes in elevation (in m) (**A**), latitude (° N) (**B**), longitude (° E) (**C**), phytoclimate (increasing values indicate warmer and drier climates) (**D**), month (**E**), and day (Julian date) (**F**) of records of the tenebrionid *Accanthopus velikensis* in Latium (central Italy) in two periods (1900–1980 and 1981–2022). Kernel densities, medians, quartiles, ranges, and outliers are represented. Results obtained with a reduced dataset.

When the analyses were replicated with the reduced dataset, records of *A. velikensis* changed their mean elevation from 393 m to 520 m (i.e., a 127 m uphill shift, which means an average shift of about 2 m per year), and the difference was marginally significant (*W* = 9099, *p* = 0.060) (Figure 3A).

The median latitude of the species records also changed from 41.892° N to 41.933° N (*W* = 8460, *p* = 0.019), which means a northward shift of about 0.04° latitude (i.e., about 5 km) in the median values (which means an average shift of about 81 m per year) (Figure 3B).

No significant change was detected for the median longitude between the first period (12.490° E) and the second period (12.403° E) (*W* = 10,563, *p* = 0.765) (Figure 3C).

Based on the multiple regression model with climatic data for 1955–1985, the thermal optimum of *A. velikensis* in the second period (i.e., for an elevation of 520 m, a latitude of 41.933° N and a longitude of 12.403° E) was 13.51 °C. This means an increase in records in the second period from localities that (in 1955–1985) were, on average, 0.7 °C colder than those of the first period, as observed for the whole dataset. Thus, using the reduced dataset, the thermal optimum of *A. velikensis* in the second period would, again, be around 14.4 °C.

*Accanthopus velikensis* also showed a significant increase in records from localities with colder phytoclimates in the second period (*W* = 12,045, *p* < 0.001) (Figure 3D), with a median value for the second period (1981–2022) corresponding to the phytoclimatic unit 8. This means that, in the second period, the species was more frequently collected in habitats characterized by vegetation of colder climates, although less profoundly than obtained using the whole dataset.

No significant change was detected for the median month between the first and the second period, which was July in both cases (*W* = 9321, *p* = 0.718) (Figure 3E). A certain shift toward earlier dates in the frequency of records was observed in the second period, as the median for the second period was, again, 7 July, but the difference was not significant (*W* = 2800.5, *p* = 0.159) (Figure 3F).

## 4. Discussion

The results obtained in this study indicate that, in the last 41 years, the tenebrionid beetle *Accanthopus velikensis* was found more frequently at higher elevations, from northern localities, and in colder and more humid habitats, compared to pre-1981 data, whereas no significant change was detected in its median phenology in central Italy.

Various studies have documented elevational range shifts in insects (especially butterflies) due to climate change [10,17,28,30,35,50,51,52,53,54,55,56,57,58,59,60]. In accordance with these previous findings, *A. velikensis* showed an increase in the median elevation from the first (1900–1980) to the second (1981–2022) period (as expected according to Prediction 1), whereas the elevational range of the species (0–1200 m) did not change between the two study periods. This pattern is consistent with one of the basic patterns identified in insect elevational shifts: a shift in average elevation with range limits unchanged [28]. The lack of change in the range limits suggests that the species continues to find suitable conditions through its whole elevational range, but its thermal optimum shifted uphill. This uphill shift is consistent with increased temperatures; in other words, because of increasing temperatures, the optimum shifted uphill, making the species more frequently found at higher elevations. The increase in the median elevation of records from the second period might be artifactual if, for any reason, higher elevational areas were more accessible in the second period than in the first one. For example, higher elevational areas might have become easier to reach by entomologists in the second period because of road development, improvements in public transportation, and increased use of personal vehicles. However, I found that the same pattern was also revealed when remote sites in the second period were excluded. Moreover, in the study region, elevation tends to increase eastwards; thus, if the increased median elevation for the second period were an effect of better exploration of eastern sectors, this would have produced an increase in the median longitude of the records, which, however, was not observed (Prediction 4). Thus, the easier accessibility of higher elevation sites does not seem to have played a substantial role (if any) in determining an increased median value of the elevation at which the species was found. Interestingly, the elevational distribution of records in the second period shows a certain concentration not only around the median, but also at very low elevations, a pattern less accentuated in the first period. This might be due to increased sampling efforts in lowland forest areas, including reserves (Circeo National Park [121], Castel Porziano Estate [119,129], Castel Fusano Park [120,122]). Using the full dataset, the elevational shift in the median value was of 157 m uphill, whereas it was of 127 m when the reduced dataset was used, which means an average uphill shift of about 2–3 m per year. Previous research on elevational shifts in insects produced variable results depending on the taxon, the study area, the considered period, and the methods used. For example, recorded velocities were up to ~22 m/year for butterflies [30] (although, in most cases, the velocities were of a few meters per year [10,17,35,50,51,52,56,58,59,60,205]); between 0.3 and 2.5 m/year for odonates [10,17]; 0.2–4.7 m/year for orthopterans [10,17]; 4–8 m/year for dung beetles [10]; and up to 0.5 m/year in ground beetles, 1.56 m/year in cerambycid beetles, 2.48 m/year in soldier beetles, and 1.3 in aquatic bugs [17]. Thus, the recorded velocity for the elevational shift observed in *A. velikensis* is within the range typically observed for other insects in a variety of contexts. However, it is important to stress that this shift indicates a higher frequency of findings at higher elevations, which does not necessarily imply a true migration, but might be due to increased abundance at higher elevations. In other words, the shift in the elevation corresponding to the species’ thermal optimum might have generated an increase in the relative abundance of individuals at higher elevations, thus increasing their probability of being found by entomologists. Although the two explanations (immigration and local increase in abundance) are not mutually exclusive, the second appears more reasonable given the low dispersal capabilities of this flightless insect.

Poleward shifts are documented in various insect groups, such as odonates [68,70], lepidopterans [50,61,62,65,66,67,69,71], orthopterans [17], hemipterans [17,63,64], and beetles (including carabid beetles, longhorn beetles, and soldier beetles) [17], and other arthropods, such as woodlice and spiders [17]. Consistent with these previous findings and our Prediction 2, records of *A. velikensis* showed an increase in the median latitude from the first (1900–1980) to the second (1981–2022) period. Using the full dataset, this result might be due to the presence of records in northern places where the species was found in the second period, whereas there were no records at similar latitudes for the first period. This translated into an ostensible range extension towards northern latitudes. While it is possible that the species has become more frequent there, it surely occurred in these areas before 1981, as it is widely distributed through the Italian peninsula (see Introduction). However, an increase in the median latitude from the first to the second period was also revealed after the exclusion of these northern records, which suggests that the species really did increase its frequency in northern areas, albeit still being present in the southern ones. As already discussed for elevation, a possible bias might be represented by an increased accessibility to northern areas during the second period, for example by improvements in road development, public transportation, and the use of private vehicles. However, while these improvements have certainly allowed entomologists to reach remote localities, there is no reason to suppose that this should have facilitated entomological exploration in northern areas more than in the southern ones. Using the full dataset, the latitudinal shift in the median value was of about 30 km poleward, whereas it dropped to 5 km when the reduced dataset was used, which means, on average, a northward shift of about 0.08–0.48 km per year. This large discrepancy reflects the high incidence of the northernmost sites from which the species was recorded in the second period, but which was not paralleled by sites at similar latitudes in the first period. Previous findings on poleward shifts in insects showed a high variability, again depending on the taxon, the study area, the considered period, and the methods used. For example, the recorded velocities were up to 1.8 km/year for butterflies [17,50]; between 1.4 and 11.5 km/year for odonates [17,70]; 0.4–1.36 km/year for orthopterans; and up to 2.2 km/year in ground beetles, 1.72 km/year in cerambycid beetles, 3.64 km/year in soldier beetles, and 4.2 km in aquatic bugs [17]. Thus, the recorded velocity for the poleward shift observed in *A. velikensis* is within the range observed for other insects in different contexts, but possibly close to the lower values. As discussed for the elevational shift, also in this case, the increase in records at higher elevations is probably due to an increase in local abundance (and, hence, detectability by entomologists) rather than migration. It is important to note that studies on poleward shifts in insects have so far been conducted at relatively large scales, while the current research suggests that effects can be deduced even at a smaller scale (the entire investigated latitudinal gradient was less than 180 km), although caution is needed in accepting this finding because of the very small geographical distance and possible biases in data collection. Although to a lesser extent compared with the elevational shift, the latitudinal shift contributed to maintaining the species in the second period more frequently in places with the same climatic conditions that characterized those in which it was more frequent in the first period (Prediction 3).

Overall, the species was found in all phytoclimatic units occurring in mainland Latium, excluding phytoclimatic units 1, 5, and 8. This is consistent with the high euryoecy of the species. Phytoclimatic unit 1 is the coldest and is restricted to the highest elevations of the region (mountain summits near the border with the Abruzzi region) out of the elevation range of the species. The lack of records from phytoclimatic units 5 and 8 is likely due to their concentration in the Sacco valley and adjacent hills (foothills of Ernici and Mainarde, Lepini, and Ausoni and Aurunci Mountains), an area from which tenebrionid records are typically rare (see map in [206]). The wide distribution of *A. velikensis* across phytoclimatic units indicates that it is a rather euryoecious species. However, the record frequencies in different units changed significantly between the two periods, with an increase in records of colder phytoclimatic units in the second period (Prediction 5). This is consistent with results concerning elevation, since colder and more humid phytoclimatic units are typical of higher elevations. The high concentration of records in phytoclimatic units 4 and 11 is a reflection of their presence on the Alban Hills, a hilly area (~300–950 m) covered with chestnut forests where the species seems to be very abundant. All of these findings indicate that *A. velikensis*, albeit euryoecious, was affected by the ongoing climate change, becoming more frequent at higher elevations and latitudes, and illustrate how climate change distinctly affects even species with broad niche widths.

Based on its distribution in Apulia, Marcuzzi [148] found that the distribution of *A. velikensis* was unrelated to the climatic conditions summarized by Lang’s pluviofactor and concluded that, being associated with subcortical spaces, which are a protective microhabitat, the species is more influenced by the microclimate than by the macroclimate. The results of the present study show that, in fact, *A. velikensis* is influenced by macroclimatic conditions and it is sensitive to changes in temperatures and precipitations. It must be stressed that subcortical spaces may only represent a relatively stable environment within the bioclimatic conditions of a certain site, and that adults are exposed to these conditions when they are active on trunk surfaces during the night.

Given the strong impact of the ongoing climate change in the Mediterranean basin [92], these results illustrate how even common and eurythermic species may be affected by climate change in this biodiversity hotspot. *A. velikensis* is a common species in terms of geographical distribution (it is widely distributed in southern Europe), ecological needs (it occupies a variety of habitats), and population size (it may be locally abundant), three criteria that are used to evaluate species vulnerability [207], yet it is not immune to the ongoing climatic change. Although *A. velikensis* continues to be frequent at lower elevations, the elevational shift observed in this species is particularly alarming, as it might be unable to efficiently track its thermal optimum because of its low dispersal capabilities. This might lead to a reduction in its lowland populations. As previous research has been mostly focused on butterflies [10,17,30,35,50,51,52,53,55,56,58,59,60,61,62,65,66,69,71], which have generally good dispersal capabilities, these findings call for more research on more sedentary insects.

Changes in phenology are one of the best known aspects of insect response to climate change [49]. For example, many insects (especially among lepidopterans) have substantially advanced their appearance [10,84,205,208,209,210,211] and extended their flight period [84,87]. Some bark beetles, butterflies, and moths have also increased the number of generations per year [86,212]. However, increased temperature may also change overwintering strategies. A delay in the winter diapause induction might lead to a complete or partial additional generation in the autumn that cannot survive or enter diapause [213,214]. As adults of *A. velikensis* are known to be present all year round, we could not expect a phenological extension, but an advance of the median activity was hypothesized (Prediction 6). However, contrary to expectations, there was no significant shift in *A. velikensis* phenology. In both periods, the species showed a unimodal phenology extending all year round and centered in July. In fact, an advance in the median day (from 22 July to 7 July) was observed, but significance was not reached. However, these results might have been influenced by the large dispersion of data, which is at least in part due to collection modalities (hand searching under bark might have led to the collection of likely overwintering individuals in winter, when the species might be not active). Data from pitfall traps and personal observations indicate that the species is mainly active between May and September. For a more reliable phenological reconstruction, careful pitfall trapping and possibly dissections to evaluate the fertility and fecundity of females and males and other parameters of the gonadal status would be helpful. A direction for future research might be to perform phenological surveys at different elevations, to also take in account the effect of the habitat features on the population density of this tenebrionid (a detail that is impossible to extrapolate from collection materials), and a space-for-time approach, in which future trajectories are inferred from contemporary spatial patterns [215,216].

In summary, the results obtained in this study supported the hypotheses that *A. velikensis* became more frequent at higher elevations (H1) and at higher latitudes (H2), but not the hypothesis that the species advanced its phenology (H3) in the second period.

## 5. Conclusions

Records of *Accanthopus velikensis* from Latium (central Italy) in the period 1981–2022 became more frequent at higher elevations and latitudes compared to their distribution in the period 1900–1980. These shifts do not seem to be imputable to an intensification of sampling at higher elevations and latitudes, but are consistent with an increase in average temperatures. By contrast, *A. velikensis* does not seem to have changed its activity pattern in response to increased temperatures, but this might be an artifact due to the inclusion of likely overwintering individuals that inflates data dispersion. The results obtained for *A. velikensis* indicate that even thermally euryoecious species can show changes in their elevational and latitudinal distribution, and that poleward shifts can be apparent even within a small latitudinal gradient.

## Figures and Tables

**Table 1:** Results of multiple regression between average annual temperature (°C) and elevation, latitude, and longitude from 106 meteorological stations in Latium (central Italy) (years: 1955–1985). *R*^2^ = 0.895, *F*_(3,102)_= 289.5, *p* < 0.00001. SE = Standard Error, *t* = Student’s t.

	Estimate	SE	*t*	*p*
Intercept	50.068	12.410	4.035	0.0001
Elevation (m)	−0.005	<0.001	−21.431	<0.00001
Latitude (° N)	−0.756	0.255	−2.963	0.004
Longitude (° E)	−0.182	0.162	−1.125	0.263

## Data Availability

Data are provided as Supplementary Materials.

## Supplementary Materials

The following supporting information can be downloaded at: https://www.mdpi.com/article/10.3390/insects15040242/s1, Figure S1: Annual average mean surface air temperature in Latium from 1901 to 2022 (data from [197]); Table S1: Characteristics of phytoclimatic units (based on [190]); Table S2: Records of *Accanthopus velikensis* from Latium; Table S3: Annual average mean surface air temperature in Latium from 1901 to 2022 (data from [197]); Table S4: Annual average mean surface air temperatures from 106 meteorological stations in Latium (elaborated from data reported in [190]).
