# Supplementary figures and images for: Upward and Poleward (but Not Phenological) Shifts in a Forest Tenebrionid Beetle in Response to Global Change in a Mediterranean Area
Source: Insects. 2024 Mar 30;15(4):242. doi: 10.3390/insects15040242 (PMC11049879; doi:10.3390/insects15040242)

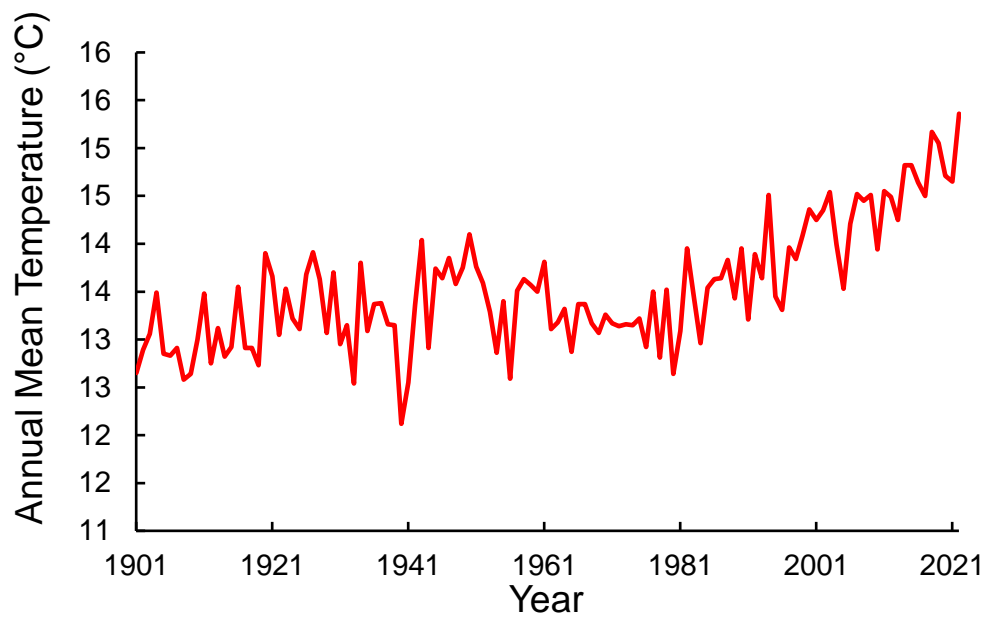

Supplement: Supplementary file 1 [file insects-15-00242-s001.zip › Figure S1.pdf]
